# The New Therapeutic Strategies in Pediatric T-Cell Acute Lymphoblastic Leukemia

**DOI:** 10.3390/ijms22094502

**Published:** 2021-04-26

**Authors:** Marta Weronika Lato, Anna Przysucha, Sylwia Grosman, Joanna Zawitkowska, Monika Lejman

**Affiliations:** 1 Student Scientific Society, Laboratory of Genetic Diagnostics, Medical University of Lublin, 20-093 Lublin, Poland; coronarysulcus@gmail.com (M.W.L.); annaprzysucha15@gmail.com (A.P.); s.grosman@op.pl (S.G.); 2 Department of Pediatric Hematology, Oncology and Transplantology, Medical University of Lublin, 20-093 Lublin, Poland; jzawitkowska1971@gmail.com; 3 Laboratory of Genetic Diagnostics, Medical University of Lublin, 20-093 Lublin, Poland

**Keywords:** T-ALL, pediatrics, novel therapies

## Abstract

Childhood acute lymphoblastic leukemia is a genetically heterogeneous cancer that accounts for 10–15% of T-cell acute lymphoblastic leukemia (T-ALL) cases. The T-ALL event-free survival rate (EFS) is 85%. The evaluation of structural and numerical chromosomal changes is important for a comprehensive biological characterization of T-ALL, but there are currently no genetic prognostic markers. Despite chemotherapy regimens, steroids, and allogeneic transplantation, relapse is the main problem in children with T-ALL. Due to the development of high-throughput molecular methods, the ability to define subgroups of T-ALL has significantly improved in the last few years. The profiling of the gene expression of T-ALL has led to the identification of T-ALL subgroups, and it is important in determining prognostic factors and choosing an appropriate treatment. Novel therapies targeting molecular aberrations offer promise in achieving better first remission with the hope of preventing relapse. The employment of precisely targeted therapeutic approaches is expected to improve the cure of the disease and quality of life of patients. These include therapies that inhibit Notch1 activation (bortezomib), JAK inhibitors in ETP-ALL (ruxolitinib), BCL inhibitors (venetoclax), and anti-CD38 therapy (daratumumab). Chimeric antigen receptor T-cell therapy (CAR-T) is under investigation, but it requires further development and trials. Nelarabine-based regimens remain the standard for treating the relapse of T-ALL.

## 1. Introduction

T-cell acute lymphoblastic leukemia (T-ALL) is a rare variant of lymphoblastic leukemia, involving heterogeneous and variable genetic abnormalities in T-lymphoid cells. This subtype tends to occur more often in adult ALL cases than in pediatric patients; the rates are 25% and 15%, respectively [1,2]. Another type of leukemia frequently diagnosed in children is precursor B-cell ALL (BCP-ALL), which accounts for 85% of childhood leukemias [3]. T-cell lymphoid leukemia has an unusual morphology and genetic and clinical features that distinguish this group from other non-T-cell cancers [4]. Its cases are characterized by male sex, leukocytosis, rapid infiltration, and a median age of 9 years. Almost 50% of patients present with a high white blood cell count (or hyperleukocytosis) [5]. Sixty percent of the patients develop mediastinal masses, and about 10% have a predisposition to central nervous system (CNS) involvement, which results in symptoms such as headaches, personality changes, vomiting, dyspnea, and visual weakness [5]. Relapses in the CNS are involved with risk treatment failure by high resistance against chemotherapy [6,7,8].

The diagnosis of childhood ALL is based on the morphology, immunophenotype, and genetic abnormalities [7]. Immunophenotyping is the standard procedure for ALL classification. The expression of CD markers is associated with the natural maturation process. Lymphoid T-cell precursors are assigned to five principal categories: pro-T EGIL T-I (cCD3+, CD7+), pre-T EGIL T-II (cCD3+, CD7+ and CD5/CD2+), cortical T EGIL T-III (cCD3+, Cd1a+, sCD3+/−), mature-T EGIL T-IV (cCD3+, sCD3+, CD1a−), and T-γ/δ [9]. The pro-T and pre-T gene expression patterns are associated with better outcomes than cortical or medullary types [5]. The current World Health Organization (WHO) classification of ALL, from 2016, incorporated a novel subgroup, early T-cell precursor ALL (ETP-ALL), which represents 15% of T-ALL cases [10]. The gene expression and immunology of this individual group were first described in 2009 by Coustan-Smith et al. [11]. 

ETP-ALL is characterized by immature precursors related to hematopoietic stem cells and myeloid progenitors [12,13,14]. Its special diagnostic criteria in immunophenotypic screening are the absence of CD1a and CD8 expression, the absence or weak expression of CD5, and the presence of at least one myeloid or stem cell marker [1,9,12]. The subtype of ETP-ALL with an elevated CD5 marker is classified as a near-ETP ALL [7]. ETP-ALL has a lower frequency of classical T-ALL genetic alterations such as *NOTCH1/FBXW7/CDKN2A* mutations [10] and a higher prevalence of *FLT3*, *NRAS*/*KRAS*, *DNMT3A*, *IDH1, IDH2, JAK3,* and *ETV6* mutations and changes associated with acute myeloid leukemia (AML) [10,15], which confirms the different genomic profile of this subgroup. Genome-wide analyses and unique genomic lesions for this new entity will be useful for developing new targeted approaches. ETP-ALL is associated with a poor prognosis. The genomic landscape of T-ALL is very wide and heterogenous. T-ALL is marked by the transcriptional activation of several protooncogenes, submicroscopic deletions of cancer suppressor genes, epigenetic deregulation, ribosomal dysfunction, altered RNA stability, cell-cycle dysregulation, and disordered signaling in the pathways *NOTCH1/FBXW7*, PI3K/Akt/mTOR, RAS/MAPK, and IL7R–JAK–STAT [7,16]. Ribosomal modifications in *RPL5, RPL10,* and *RPL11*, and plant homeodomain factor gene *PHF6* are relatively newly detected changes that involve chromatin modification [7]. The activation of *NOTCH1* signaling was first described in 2004 in more than 50% of pediatric patients, the most common mutation, which became a hallmark of T-ALL [17]. However, the prognostic value of *NOTCH1* mutation has been questioned. The genetic alterations in pediatric T-cells include the monoallelic deletion of 17q12, involving the tumor suppressor *NF1* and also *MYB* in children under 2 years old as a result of the activation of the t(6;7) translocation or duplications or the amplification of 6q23 [7]. The activation of oncogenic transcription factors including the basic helix-loop-helix genes (bHLH) *TAL1, TAL2, LYL1*, and *OLIG2* (BHLHB1); *TLX1* (HOX11), *TLX3* (HOX11L2), *NKX2-1, NKX2-2, and NKX2-5*; the LIM-only domain genes *LMO1* and *LMO2* is also a hallmark of T-ALL. Subtype TLX3 (T-cell leukemia homeobox protein 3) characterizes the lack of a functional T-cell receptor (TCR) or presence of γ/δ TCR, rearrangements of the transcription factor *TLX3*, exists in 25% T-ALL, and indicates favorable outcomes. The TLX1/NKX2.1 (T-cell leukemia homeobox protein 1/NK2 homeobox 1) subtype presents in 10% T-ALL with genomic rearrangements involving either *TLX1* or *NKX2.1*, CD1 expression, and differentiation arrest at the cortical stage; it is a proliferative subtype, and it correlates with excellent outcome in patients. Another molecular subtype is TAL/LMO (transcription activator-like/LIM domain-only), which is related to poor outcome. The ectopic expression of *TAL1, TAL2, LYL1, LMO1, LMO2,* or *LMO3* and late cortical immunophenotype, mutations of the PI3K signaling pathway (*PTEN* and *PIK3R1*), *USP7* alterations, *LEF1* deletions, and *SIL-TAL1* fusion are features described as characteristic in this subtype. Some point mutations seen in the *TAL1* gene lead to the overexpression of this gene, and these mutations cause the silencing of target genes encoding for E47 and E12 variants of E2A transcription factors. Although these genetic alterations significantly contribute to and are related to treatment outcome, none of them is presently used for risk stratification in T-ALL [10,17]. 

Other genetic anomalies have been identified in ETP-ALL. The mutations are divided into three groups: hematopoietic development (*IKZF1, ETV6, RUNX1, GATA3,* and *EP300*), *MAPK* and cytokine receptor signaling (*NRAS, KRAS, IL7R, JAK1, JAK3, PTPN11*, *NF1,* and *SH2B3*), and chromatin-modifying genes (*EED, EZH2, SUZ12,* and *SETD2*). The inactivation by the mutation or deletion of *ETV6, RUNX1,* and *GATA3* has also been described in AML and correlates with poor outcomes in ETP-ALL [10]. 

Sequencing can identify the common genetic alterations in childhood T-ALL, but the prognostic value of these identified genetic lesions remains unknown [10]. To estimate the prognostic value of these mutations and realize therapeutic stratification according to them, we need more multicenter, randomized clinical trials. The most important predictor for outcomes in T-ALL patients is still the minimal residual disease (MRD) level [6,10]. The contemporary regimen treatment for T-ALL is based on chemotherapy, steroids, and allogeneic hematopoietic stem cell transplantation (all-HSCT). In the last 20 years, the use of aggressive cytoreduction has improved survival rates, which are now similar to those for BCP-ALL [6,10]. However, relapses remain a therapeutic problem. Unlike for BCP-cell ALL, the prospects for treating relapsed childhood T-ALL are dismal due to the more biologically heterogeneous characteristics of the relapse clones: the 5-year overall survival and event-free survival rates in cases of relapse are about 25% [2,10,18]. Therefore, a new look at the wide genetic diversity of T-ALL lesions is important for elaborating innovative therapeutic options, identifying genetic subgroups for stratifying treatments, and predicting the outcomes. 

This review summarizes the current treatment and promising novel, emerging strategies.

## 2. Treatment of T-Cell Acute Lymphoblastic Leukemia

Although, historically, the outcomes in T-ALL were poorer than those in B-ALL for children, contemporary intensive care has made the prognosis of T-ALL similar [10,19,20]. Patients can be classified into appropriate risk groups based on the risk of treatment failure. Patients with newly diagnosed T-ALL are currently treated with intensive chemotherapy, which has been occasionally supplemented with cranial radiotherapy (CRT) [10,19]. To avoid the development of resistance, different treatment blocks of chemotherapy are implemented and divided into phases: induction, consolidation and reduction [6]. Patient stratification depends on many biological factors, including the response to the initial treatment, and the assessment of the MRD [6]. The MRD remains the most important factor determining the prognosis in children with T-ALL [6,10]. The MRD can be deduced by flow cytometry (FCM-MRD) and real-time polymerase chain reaction (PCR-MRD). The Children’s Oncology Group (COG) has presented a risk classification for T-ALL based on MRD at different time points (TP), in which three risk groups are distinguished: the standard risk group (on Day 29, MRD <0.01%), intermediate risk group (on Day 29, MRD <0.01%; end of consolidation, MRD <0.1%), and very high-risk group (MRD at the end of consolidation ≥0.1%) [10]. 

Patients with T-ALL treated according to the protocol Associazione Italiana di Ematologia Oncologia Pediatrica-Berlin-Frankfurt-Münster (AIEOP-BFM) 2017 are stratified based on the MRD assessment (FMC-MRD and PCR-MRD), the response to initial steroid therapy and complete remission on Day 33. The patients are classified into the early standard risk group (SR) if the FCM-MRD in the bone marrow on Day 15 < 10%, there is a good response to prednisone, and the PCR-MRD on Day 33 shows negativity for all the investigated markers or at least one marker has a quantitative range ≤ 10^−4^ and there is complete remission on Day 33. A poor response to prednisone, an FCM-MRD in the bone marrow on Day 15 ≥ 10%, a lack of complete remission on Day 33, and a PCR-MRD at TP2 ≥ 5 × 10^−4^ (12 weeks of therapy) puts patients into the high-risk group (HR). Those with no HR criteria are classified as non-high-risk (non-HR). The MRD at the end of induction and at the end of consolidation has been proven to be an independent factor useful for predicting the outcomes and in the stratification of patients to the appropriate risk groups [6,10,19]. If the patients are positive for MRD at the end of induction but negative at the end of consolidation, they have a good prognosis, with a 7-year EFS of 80.6% with conventional chemotherapy. In a randomized clinical trial, AIEOP BFM 2017, for the treatment of children with newly diagnosed ALL, the MRD is evaluated in TP1 on Day 33 at the end of the induction phase, and in TP2 after 87 or 92 days after the consolidation phase [10,19]. The AIEOP BFM 2000 study showed that T-ALL patients with MRD <10^−4^ on Day 78 had similar results regardless of the MRD on Day 33. The United Kingdom Acute Lymphoblastic Leukemia (UKALL) 2003 study indicated that allo-HSCT should be recommended in patients with an end-induction MRD ≥5%, and this consolidation therapy now includes nelarabine treatment in patients with HR [19]. 

Many clinical trials have reported an improvement in the prognosis of patients with T-ALL through early treatment [19]. The first step of chemotherapy is a remission induction lasting 4–6 weeks. Vincristine, corticosteroids, asparaginase, and additionally, the anthracyclines are the drugs used during the induction [6]. The UKALL 2003 study reported that low-intensity 3-drug induction and low-intensity consolidation resulted in worse responses in low-risk patients than four-drug treatment in high-risk patients, with the results showing event-free survival (EFS) of 80.1% and 86.7%, respectively. These conclusions were confirmed by comparing treatment with CCG-1952 and CCG-1991, where worse results were obtained during the 3-drug treatment [10,19]. In determining the intensity of treatment, the different corticosteroid treatment regimens that inaugurate the treatment should be considered. The differences are in the use of dexamethasone or prednisone. Dexamethasone reduces the incidence of relapses by increasing central nervous system (CNS) strength and penetration; however, the disadvantage is a higher rate of infectious toxicity, including avascular necrosis [6,10,19]. The randomized clinical trial AIEOP BFM 2000 comparing the effect of 21 days of dexamethasone at a dose of 10 mg/m^2^ per day with that of prednisone at a dose of 60 mg/m^2^ per day showed higher toxicity and mortality in the dexamethasone-treatment group (2.5% vs. 0.9%). However, there were fewer relapses in the dexamethasone group, and the 5-year risks of relapse were 10.8% and 15.6%, respectively. In the DFCI ALL 00−01 protocol, the 5-year EFS for the dexamethasone group was 96% vs. 65% for the prednisone group. 

Nelarabine is a new agent that was initially used for patients with relapsed/refractory T-cell ALL, but it is currently also used for newly diagnosed T-ALL. Nelarabine is an antimetabolite, a water-soluble pro-drug of arabinosylguanine nucleotide triphosphate, a purine deoxyguanosine analog, leading to the inhibition of DNA synthesis [21]. A dose of 650 mg/m^2^/day for 5 days has been used and proved to be optimal for children [18]. The side effects of nelarabine include central or peripheral neuropathies, dizziness, confusion, ataxia, seizures, mood alterations, and hematologic events—neutropenia, anemia, and thrombocytopenia. [22] 

COG AALL0434, conducted from 2007 to 2014, was the largest trial ever conducted for newly diagnosed T-ALL. The trial enrolled 1895 patients aged 1–31, using an augmented Berlin–Frankfurt–Muenster (ABFM) regimen [21,23]. The patients were randomized to receive Capizzi-escalating dose methotrexate without leucovorin rescue plus pegasparagase (C-MTX) or high-dose MTX (HDMTX) plus leucovorin rescue. Intermediate- or high-risk patients were randomly assigned to receive or not six five-day courses of nelarabine at 650 mg/m^2^/d; low-risk patients were not regarded for nelarabine randomization. The 5-year disease-free survival (DFS) for nelarabine versus no nelarabine in the randomized cohorts was 88.2% vs. 82.1%, respectively. The 5-year overall survival (OS) was also better for patients assigned to receive nelarabine (90.3%) than for those not assigned (87.9%). C-MTX with nelarabine was the most effective, and the DFS was 91.4%. The HDMTX regimens were also improved by the addition of nelarabine. A decrease in CNS relapse was noticed. The authors reported that nelarabine improves the outcomes for children and young adults with T-ALL, especially with high-risk disease, without differences in age or race groups. [21,23] 

HSCT is not needed to cure most children. It is recommended to consider HSCT from an appropriate donor in children who, at the end of consolidation, have high MRD (in the US, >0.1%, and in the UK, >0.05%). The UKALL2003 study reported that HSCT is recommended for patients with MRD ≥5% at the end of induction. This proposition does not concern patients under 16 years of age in remission at the end of consolidation therapy [19]. 

ETP-ALL was initially thought to have a very poor prognosis, but the opinions on it vary. Vadillo et al. reported that patients with ETP-ALL had an overall survival (OS) of 19%, compared to the 84% rate for other T-ALL subtypes [12,14]. No difference in OS was observed in the COG AALL0434 study and UKALL 2003 trial. Moreover, the OS in the COG trial was higher in the ETP and near-ETP group, at 93% and 91.6%, than in the non-ETP groups [10]. In addition, the clinical study AALL0434 reported that for ETP-ALL, only 18.6% of the subjects had MRD <0.01% on Day 29, and for non-ETP-ALL, MRD <0.01% was observed in 69.5%. The induction failure rates were 7.8% and 1.1%, respectively. Despite the risk of occurrence and the differences in MRD, it is recommended that these patients are treated the same and according to the same recommendations as for non-ETP-ALL. By contrast, the UKALL 2003 study reported non-significantly inferior outcomes in patients with ETP-ALL, and the 5-year EFS was 76.7% vs. 84.6%, respectively [10,19,20]. The optimal treatment regimen for ETP-ALL remains uncertain. Due to the conflicting data of these clinical trials, further studies are necessary. 

The main cause of relapse is the high rate of chemotherapy resistance initiated by novel mutations and the early infiltration observed in T-cell ALL. Contemporary studies have shown that 80% of relapses occur within 2 years of diagnosis [7]. Relapses usually occur in the bone marrow (57.3%), central nervous system (20.9%), and testis (5.3%). A combined bone marrow relapse (13.5%) is diagnosed with more than ≥5% blasts with the presence of other extramedullary localization [24]. A CNS-isolated relapse is usually followed by bone marrow relapse, and it is recognized when the white blood cell (WBC) count in the cerebrospinal fluid (CFS) is more than >5/µL, with the presence of blasts [25,26]. Children with relapsed T-cell ALL clearly present a worse prognosis than patients with BCP-ALL, because the survival rate for T-ALL relapse is lower than 25% [19,27]. The current protocols of relapse treatment include rotating multi-drug chemotherapy cycles or continuous cytoreduction followed by allogeneic HSCT. Achieving a second complete remission with chemotherapy alone in reinduction schema is more difficult in children with relapsed T-cell acute lymphoblastic leukemia [10,27]. Moreover, relapse cells also acquire mutations that are novel with respect to those found at diagnosis [28]. The reinduction approach in T-ALL is not globally standardized, but the regimens are usually based on four drugs that are used in primary induction such as vincristine, steroids, asparaginase, and an anthracycline [3]. To improve the poor survival rates, more recent ALL relapse trials have allocated patients to randomizations involving novel agents and therapies, to achieve remission before allo-HSCT [24]. A combination of nelarabine, etoposide, and cyclophosphamide in the reinduction led to 44% remission rates for the first relapse of T-ALL [10]. In AIEOP BFM ALL 2017, based on the International Study for Treatment of Childhood Relapsed High-Risk ALL 2010 (IntreALL-HR) protocol, patients with T-ALL are receiving treatment in the induction phase without or with randomization to bortezomib. Bortezomib is a selective inhibitor of the 26S proteasome. The anti-cancer mechanisms of bortezomib are the result of the upregulation of proapoptotic proteins (NOXA), the suppression of the NFκB signaling pathway, and the inactivation of anti-apoptotic proteins (*Bcl-XL, Bcl-2,* and *STAT3*) [29]. The most important mechanism in T-cell ALL is associated with the ability to inhibit the *NF-κB* and *NOTCH1* signaling pathways [30]. Bortezomib increases the activity of the multiagent cytoreduction treatment in polytherapy and sensitizes the malignant cells to corticosteroids. As a single agent, bortezomib did not have a significant effect. Horton et al. reported that adding this drug to standard reinduction chemotherapy with vincristine, doxorubicin, pegaspargase, and prednisone improved the response rates in patients with T-ALL, and second complete remission (CR2) was achieved in 68% of the patients. Another combination of bortezomib with mitoxantrone, dexamethasone, vincristine, and pegaspargase is also effective in reinduction schema [31]. In the ALLR3 trial, which was conducted in the UK for the treatment of relapsed ALL, children received mitoxantrone with dexamethasone, methotrexate, and cytarabine in three blocks. The use of mitoxantrone resulted in a 65% 3-year progression-free survival rate. Nonetheless, for T-ALL relapse, hematopoietic cell transplantation is the most successful method and is usually necessary after achieving a second remission [30]. In AIEOP BFM ALL 2017, based on the InterALL 2010 HR protocol, all high-risk (HR) patients are candidates for allogeneic stem cell transplantation (SCT). This favorable outcome for non-sibling donor SCT is reported only in the high-risk patient group, not in standard-risk patients. In the United Kingdom, allo-HSCT is also used in early CNS relapses and in patients with late combined CNS relapses and high MRD levels (10^−4^ cells or more at the end of the induction phase). For patients with early isolated extramedullary relapse, the benefits of HSCT are questionable. Secondary and tertiary relapses have extremely poor survival rates, at 9% and 6%, respectively [32]. The prognosis is worse for a patient with relapse after allo-HSCT treatment or with T-cell ALL. This patient has a small possibility of surviving after intensive cytoreduction treatment followed by allo-HSCT [33]. 

## 3. Novel Approach in T-ALL Treatment

Conventional chemotherapy is marked by high efficacy, although 15–20% of children with T-ALL develop relapse, which is characterized by lower cure rates [33]. A new approach, including monoclonal antibodies and chimeric antigen receptor (CAR) T-cell therapy, has been introduced in B-ALL treatment. This approach is considered to avoid the toxicity caused by chemotherapy, by focusing on the molecular pathophysiology of the disease. The study of recurrent mutations is significant for alternative treatment strategies for individual cases. It is hoped that identifying genetic markers could predict outcomes more precisely.

**Figure 1:**
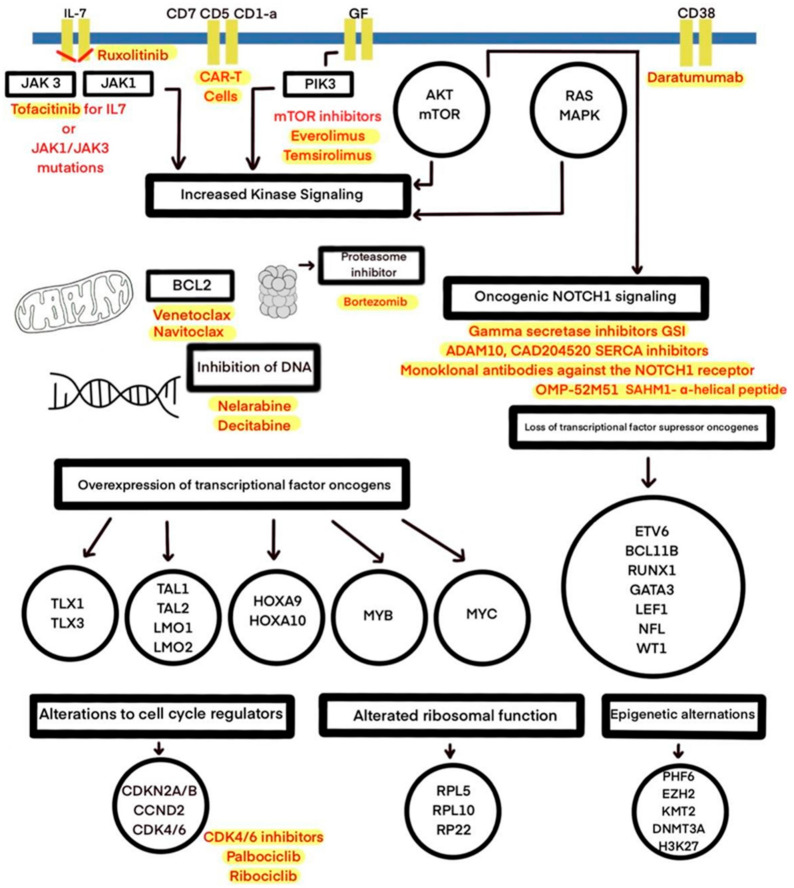
The molecular therapeutic targets used in preclinical and clinical studies of T-ALL treatment.

Sequencing efforts have revealed several genetic alterations in transcription factors and signaling pathways, as well as epigenetic alterations, mistranslations, and the alteration of RNA stability and are shown in Figure 1 and Table 1 [34]. The most frequent abnormality in T-cell ALL involves *NOTCH1* mutations. Originally, such mutations were found in approximately 50% of cases; in current studies, these mutations occur in over 60% of cases, reaching 75% in recent reports. [2,5,14,20,34]. The Notch1 signaling pathway is crucial in the thymus for early T-cell lineage specification, proliferation, and development, and it can be dysregulated through activating mutations (first identified through the finding of a rare chromosomal translocation t(7;9)(q34;q34.3) [2,20]. In T-ALL, constitutive *NOTCH1* activation, induced by receptor mutations, results in a lack of T-cell development and activates cell growth and metabolism genes [14]. Moreover, the NOTCH1 pathway has a central driver role in T-cell metabolism and promotes leukemia cell growth through the direct upregulation of anabolic pathways: ribosome biosynthesis, protein translation, and nucleotide and amino acid metabolism [7]. *NOTCH1* can also be activated as a result of the dysregulation of other pathways—c-myc and *PI3K/AKT/mTOR* [20]. In 10–15% of cases, the aberrant activation of the *NOTCH* pathway occurs through distinct mechanisms, such as a loss of function of the negative regulator *FBXW7*. FBXW7 is a protein promoting *NOTCH1* proteasomal degradation, and it leads to increased *NOTCH1* protein stability [7,20]. The high prevalence of mutations in the *NOTCH* signaling pathway in T-ALL has led to the development of therapies aimed at the inhibition of *NOTCH* signaling, including γ-secretase inhibitors (GSIs), soluble notch proteins, and inhibiting peptides [2,20]. GSIs are small molecules that cause a systemic block of all four *NOTCH* receptors, leading to the inhibition of *NOTCH* signaling [35]. Due to their features, GSIs inhibit cell growth by inducing cell cycle arrest at the G0–G1 phase and induce apoptosis in certain T-ALL cell lines [36]. Early trials presented limited success, as the original use of GSIs was limited to Alzheimer’s disease treatment [14,35]. The main obstacle for GSI development in the context of T-ALL therapy, apart from a limited antileukemic effect, is gastrointestinal toxicity. In the Dana-Farber Cancer Institute’s phase I clinical trial testing MK-0752, the most frequent side effect was grade 3/4 diarrhea, as a result of the inhibition of *NOTCH1* and *NOTCH2* in the intestine [35,37]. Moreover, several mechanisms of GSI resistance were noticed [36]. The current aim is to devise a combined therapy that can overcome GSI resistance, improve the antileukemic response, and reduce the side effects resulting from the inhibition of Notch signaling [36]. Research on PF-03084014, an oral, non-competitive, reversible GSI developed by Pfizer, demonstrates that by intermittent dosing schemes, there is a possibility of reducing toxicity, because the systemic side effects induced by GSIs seem to be time- and dose-dependent [35,36]. The preclinical analysis of PF-03084014 and glucocorticoids in combination shows a synergistic antileukemic effect and decreased intestinal toxicity due to protective glucocorticoid activity [35,38]. Other preclinical studies have suggested the synergistic activity of withaferin A, rapamycin, and vorinostat in combination with GSIs in vitro [39]. Additionally, a combination of GSIs and chloroquine has presented a synergistic effect in vitro on the T-ALL cell line and is associated with fewer side effects [40]. Using GSIs as a single agent seems to be insufficient in T-ALL carrying *Notch1* mutations, and combined treatments, with reduced gastrointestinal toxicity and enhanced antileukemic effects, may be alternative options [41]. 

Apart from the limitations and constant improvements in GSI therapy, distinct approaches targeting the *NOTCH* signaling pathway have been developed, including *ADAM10* and *CAD204520* SERCA inhibitors. Mastermind-inhibiting peptide—the α-helical *SAHM1*—is under active study. In addition, monoclonal antibodies against the *NOTCH1* receptor have shown efficacy in preclinical trials. OMP-52M51, a monoclonal antibody produced by mice immunized by human NOTCH1 protein fragments, showed antitumor effects in a T-ALL cell line in vitro and in vivo in xenograft models [41,42]. 

Targeting the dysregulation of the cell cycle via CDK4/CDK6 is a different prominent approach in T-ALL treatment [7]. CDK4/6 inhibitors are showing promise in clinical trials. A phase I trial (ClinicalTrials.gov identifier: NCT03515200) has investigated palbociclib added to other chemotherapeutic drugs such as dexamethasone in participants aged up to 21 years. Another clinical trial (ClinicalTrials.gov identifier: NCT03132454), still recruiting patients 15 years and older with relapsed/refractory (R/R) leukemia, is studying a combination of palbociclib with sorafenib, decitabine, or dexamethasone [22]. A phase I trial (ClinicalTrials.gov identifier: NCT03792256) is studying palbociclib with a standard reinduction chemotherapy in children with R/R ALL and lymphoblastic lymphoma (LL). Clinical trials including children with T-ALL are presented in Table 2.

CDK4/6 inhibitors cause cell-cycle arrest in T-ALL cells, which can interfere with conventional chemotherapy outcomes, because cytotoxic chemotherapeutic agents rely on cell proliferation. Pikman et al. evaluated the CDK4/6 inhibitor LEE011 (ribociclib) and combined it with standard chemotherapeutic agents used to treat ALL. LEE011 was found to be antagonistic with respect to mercaptopurine, methotrexate, doxorubicin, or l-asparaginase and synergistic with glucocorticoids and mTOR inhibitors, which warrants further investigation [22,43]. 

Canté-Barrett et al.’s study on 146 pediatric T-ALL cases showed that 49% of the patients harbored at least one mutation in the JAK–STAT, PI3K–AKT, or RAS–MAPK pathways, underscoring the role of the activation of those pathways for leukemogenesis [44]. 

The IL-7R/JAK/STAT pathway is important for T-cell development and homeostasis, promoting cell survival. The systematic screening of T-ALL genomes has revealed activating mutations in *IL7R*, *JAK1*, *JAK3*, and/or *STAT5* in 20–30% of T-ALL cases, with frequent occurrences in ETP-ALL cases [45]. The inhibition of *JAK1/2* by using ruxolitinib has been effective in primary xenograft models of ETP in both the presence and absence of JAK–STAT mutations and lessened the hyperactivation effect of IL-7 [2,14,46]. Preclinical studies have also demonstrated the activity of tofacitinib for T-ALL cells with IL7R or JAK1/JAK3 mutations [42]. 

The activation of the PI3K/Akt/mTOR signaling pathway is caused by inactivating mutations or deletions in the *PTEN* tumor suppressor gene, which is observed in 10% of T-ALL. Some T-ALL cases show gain-of-function mutations in the regulatory and catalytic subunits of *PI3K* [14,42]. Aberrant activation leads to improved cell growth, metabolism, and proliferation as well as limited apoptosis, and it can contribute to glucocorticoid resistance [22,42]. Clinical trials are evaluating the mTOR inhibitors everolimus and temsirolimus [22]. A phase I/II trial (ClinicalTrials.gov identifier: NCT00968253) tested the safety and efficacy of everolimus in combination with HyperCVAD chemotherapy in relapsed/refractory acute lymphoblastic leukemia; the therapy appeared to be moderately effective but well-tolerated [47]. A phase I study (ClinicalTrials.gov identifier: NCT01523977) aimed to learn more about how everolimus works in combination with other drugs commonly used to treat relapsed ALL—prednisone, vincristine, PEG-asparaginase, and doxorubicin. The results showed that this combination was feasible [48]. A phase I study (ClinicalTrials.gov identifier: NCT03740334), currently recruiting, is testing ribociclib in combination with everolimus and dexamethasone in relapsed ALL among patients 1–30 years old. 

The *ABL1* gene encodes a tyrosine kinase, and its activation is observed in up to 8% of T-ALL cases [2,42]. The most frequent *ABL1* rearrangement is NUP214–ABL1 fusion between the nuclear pore complex NUP214 and the kinase ABL1, which was described in T-ALL cell lines and found together with *TLX1* or *TLX3* expression [2,22,42]. In human NUP214-ABL1-positive T-ALL cell lines, the tyrosine kinase inhibitors imatinib, dasatinib, and nilotinib were shown to have activity and induced apoptosis, which could provide clinical benefit in some cases [2]. 

The antiapoptotic protein BCL-2 (B-cell lymphoma 2), essential for proper hematopoiesis, has become a novel approach for targeted therapies in ALL. Compounds targeting prosurvival BCL-2 family members have been under investigation. Several BH3 mimetics, triggering apoptosis, have been tested [49,50,51]. Chonghaile et al. used the BH3 profiling of primary samples and cell lines to determine antiapoptotic protein dependencies in T-ALL. The study showed that the dependence on BCL-2 or BCL-XL was determined by the maturation stage of the malignancy. The in vivo and in vitro studies suggested that T-ALL is largely BCL-XL dependent, but that samples with an early T-cell progenitor phenotype are more BCL-2 dependent. The apoptotic sensitivity to the BH3 mimetics ABT-263 and ABT-199 also demonstrated dependence according to the differentiation stage of the leukemia, showing the selective sensitivity of ETP-ALL to ABT-199 and suggesting a potential effective drug for that treatment-resistant subgroup [50]. The lack of selectivity of ABT-737 for BCL-2, BCL-XL, and BCL-W (and its orally bioavailable derivative, ABT-263 navitoclax) limited its clinical development due to severe thrombocytopenia resulting from BCL-XL inhibition in megakaryocytes. To overcome this limitation, ABL-199 (venetoclax) was developed. Venetoclax is a small molecule, BCL-2-selective BH3 mimetic that spares platelets [49,51,52]. The Food and Drug Administration (FDA) approved venetoclax for the treatment of chronic lymphocytic leukemia (CLL) and newly diagnosed AML in adults >75 years in combination with other drugs [49,53]. Venetoclax offers promising potential therapeutic benefits and antileukemic activity, although the possibility of resistance may limit the use of this drug as a single agent [54,55]. The combination of ABT-199 and chemotherapeutic agents or other targeted therapies could increase the chemosensitivity of leukemic cells, protect against resistance, and decrease dose-dependent chemotherapeutic side effects [51]. Venetoclax has shown notable clinical activity in patients with T-ALL in combination with chemotherapy [53,56], decitabine [53], nelarabine [57], and bortezonib (VEBO) [55], especially for patients with R/R T-ALL. A phase I study (ClinicalTrials.gov identifier: NCT03236857) to evaluate the safety and pharmacokinetics of venetoclax and to determine the dose-limiting toxicity is recruiting pediatric and young adult patients with relapsed or refractory ALL. An early-phase dose-escalation study (ClinicalTrials.gov identifier: NCT03181126) of venetoclax in combination with navitoclax and chemotherapy is also ongoing for children (≥4 years old) and adults with R/R ALL. 

Cellular and antibody-based therapies have shown efficacy and significantly improved outcomes for patients with B-cell malignancies [58,59]. Chimeric antigen receptor (CAR) T-cells have been approved by the US Food and Drug Administration (FDA) for the treatment of relapsed/refractory B-cell acute lymphoblastic leukemia (B-ALL) and diffuse large B-cell lymphoma (DLBCL) [59]. Despite the great development of targeted cellular therapies, T-lineage neoplasms remain a challenge for CAR-T cells due to the limited ability to distinguish between therapeutic, normal, and malignant T-cells. Target antigens shared between T effector cells and T-cell malignancies leads to CART self-targeting, called fratricide, and to profound T-cell aplasia induced by the destruction of normal T-cells, leading to life-threatening opportunistic infections [22,59,60,61,62]. Moreover, there is a risk of the contamination of CAR-T-cell products with malignant T-cells [59]. CD7, CD5, and CD1a- targeted CAR-T cells have been developed and shown therapeutic potential. They demonstrated limited fratricide and antileukemic activity in vitro and in vivo in xenograft models [1,61,62,63]. Fratricide-resistant, universal, “off-the-shelf” CAR-T cells targeting CD7 (or UCART7), generated through CRISPR/Cas9 gene editing, leading to a lack of expression of both CD7 and the T-cell receptor alpha chain, have also shown promising preclinical results [22,60]. A phase I trial of CD5-targeted CAR-T cells for patients with T-cell malignancies is under investigation (ClinicalTrials.gov identifier: NCT03081910). 

Daratumumab is a human immunoglobulin G1κ monoclonal antibody that binds to a specific epitope of CD38, showing efficacy in relapsed multiple myeloma therapy [64,65]. Moreover, daratumumab has been the most promising antibody-based approach in T-ALL treatment [22]. Blasts collected from children and young adults with de novo T-ALL demonstrated the surface expression of CD38, which remained stable after 1 month of multiagent chemotherapy. The antileukemic efficacy was tested with patient-derived xenografts (PDX), showing potential for further research [64]. Currently, daratumumab in addition to standard chemotherapy is under investigation in a phase II trial for pediatric and young adult participants with relapsed and/or refractory T- or B-cell ALL (ClinicalTrials.gov identifier: NCT03384654). 

## 4. Conclusions

Some of the pathways affected by the genetic aberrations might have relevance as therapeutic targets in selected groups of pediatric T-ALL patients. Novel therapies targeting molecular aberrations offer promise for achieving better first remission, with the hope of preventing relapse. The employment of a precisely targeted therapeutic approach is expected to improve the cure of the disease and quality of life of patients. 

## Figures and Tables

**Table 1:** Promising targets and molecules in novel T-ALL therapies.

Target	Molecules	References
NOTCH signaling pathway	GSIs: γ-secretase inhibitors	[2,20,35,41,42]
	ADAM10 and CAD204520 SERCA inhibitors	
	SAHM1: α-helical peptide	
	OMP-52M51: monoclonal antibody	
CDK4/6 kinases	palbociclib	[7,22]
	ribociclib	
IL-7R/JAK/STAT signaling pathway	ruxolitinib	[2,14,42,45,46]
	tofacitinib	
PI3K/Akt/mTOR signaling pathway	everolimus	[14,22,42]
	temsirolimus	
ABL kinase	imatinib	[2,22,42]
	dasatinib	
	nilotinib	
BCL-2 protein	venetoclax	[49,50,51]
	navitoclax	
CD5, CD7	CD7, CD5, and CD1a- targeted CAR T-cells	[1,61,62,63]
CD38	daratumumab	[22,64]

**Table 2:** Selected clinical trials including children with T-ALL.

Target	Molecule	Patients	ClinicalTrials.gov Identifier/References
CDK4/6	palbociclib+chemotherapy	Up to 21 years old with R/R ALL	NCT03515200 [22]
	palbociclib+sorafenib, decitabine, dexamethasone	15 years old and older with R/R leukemia	NCT03132454 [22]
	palbociclib+chemotherapy	12 months to 31 years old with R/R ALL or LL	NCT03792256
	ribociclib+everolimus, dexamethasone	1 to 30 years old with R/R ALL	NCT03740334
PI3K/Akt/mTOR	everolimus+HyperCVAD chemotherapy	10 years old and older with R/R ALL	NCT00968253 [47]
	everolimus+prednisone, vincristine, PEG-asparaginase, doxorubicin	18 months to 21 years old with R/R ALL	NCT01523977 [48]
BCL-2	venetoclax+chemotherapy	Up to 25 years old with R/R ALL	NCT03236857
	venetoclax+navitoclax. chemotherapy	4 years old and older with R/R ALL or LL	NCT03181126
CD5	CAR T cells targeting CD5	Up to 75 years old with T-cell malignancies	NCT03081910
CD38	daratumumab+chemotherapy	1 to 30 years old with	NCT03384654

## Data Availability

No new data were created or analyzed in this study. Data sharing is not applicable to this article.

## Abbreviations

AKT1	serine-threonine protein kinase
Akt	protein kinase B
ALL	acute lymphoblastic leukemia
AML	acute myeloid leukemia
B-ALL	B-cell acute lymphoblastic leukemia
BCL-2	B-cell lymphoma 2
BCL-XL	B-cell lymphoma-extra large
BCP-ALL	B-cell precursor acute lymphoblastic leukemia
CNS	central nervous system
COG	The Children’s Oncology Group
CRT	cranial radiotherapy
CR2	second complete remission
DFS	disease-free survival
DLBCL	diffuse large B-cell lymphoma
EGIL	The European Group for the Immunologic Classification
EFS	event-free survival
ETP-ALL	early T-cell precursor ALL
FBXW7	F-Box And WD Repeat Domain Containing 7
FCM	flow cytometry
FDA	Food and Drug Administration
GSI	γ-secretase inhibitors
HR	high risk
HSCT	hematopoietic stem-cell transplantation
IL7R	interleukin-7 receptor
JAK 1	janus kinase 1
LBL	acute lymphoblastic lymphoma
MAPK	mitogen-activated protein kinases
MRD	minimal residual disease
mTOR	mechanistic target of rapamycin
MTX	methotrexate
OS	overall survival
PCR	polymerase chain reaction
PDX	patient-derived xenografts
PIK3	phosphoinositide 3-kinase
SERCA	sarco/endoplasmic reticulum Ca^2+^-ATPase
SR	standard risk
STAT	signal transducer and activator of transcription
T-ALL	T-cell acute lymphoblastic leukemia
TLX1/3	T-cell leukemia homeobox protein ⅓
TP	time point
WBC	white blood cell
T-ALL	T-cell acute lymphoblastic leukemiatime point
TLX1/3	T-cell leukemia homeobox protein ⅓
TP	time point
WBC	white blood cell
